# Severe Obesity-Induced Computed Tomography Restrictions: A Case of Renal Infection With Bacteremia

**DOI:** 10.7759/cureus.79322

**Published:** 2025-02-19

**Authors:** Takuya Kitamura, Hidenari Nomura, Kohei Fukutomi, Masahito Ogura

**Affiliations:** 1 Department of Endocrinology and Metabolism, National Hospital Organization Kyoto Medical Center, Kyoto, JPN; 2 Diabetes Center, National Hospital Organization Kyoto Medical Center, Kyoto, JPN; 3 Department of Endocrinology, Metabolism, and Hypertension Research, Clinical Research Institute, National Hospital Organization Kyoto Medical Center, Kyoto, JPN; 4 Department of General Internal Medicine, National Hospital Organization Kyoto Medical Center, Kyoto, JPN

**Keywords:** bacteremia, diabetes, imaging limitations, proteus mirabilis, severe obesity

## Abstract

Medical equipment has weight limits, but healthcare staff is often unaware of their exact specifications. However, as obesity rates rise globally and international mobility increases, healthcare providers will be required to treat more patients with extremely high body weight. We report a rare case of a 60-year-old, 224.8 kg man with diabetes who presented with a fever but could not initially undergo a computed tomography (CT) scan due to the scanner weight limit of 204 kg.The patient was diagnosed with a urinary tract infection (UTI), based on the detection of *Proteus mirabilis* from both blood and urine cultures and the fact that UTIs are the most common clinical manifestation of *P. mirabilis.* Treatment with antibiotics was performed without excluding conditions requiring surgical intervention among UTIs due to the inability to perform CT scans. On day 54 (post-discharge), his weight had decreased to 199 kg, allowing for a CT scan that revealed an enlarged left kidney and increased perirenal fatty tissue accumulation. The imaging findings, along with the gradual improvement in C-reactive protein levels and absence of fever, indicated a healed renal infection. This case underscores the impact of severe obesity on healthcare delivery. Healthcare staff should be familiar with actual weight or size limits for the available medical equipment. Contrary to assumptions, magnetic resonance imaging may be a viable alternative in some facilities for patients unsuitable for CT. Additionally, healthcare staff should be made aware of the availability of high-capacity imaging equipment in nearby hospitals. In conclusion, healthcare staff should be prepared in advance for cases in which patient weight exceeds medical equipment limits.

## Introduction

The worldwide prevalence of obesity, adjusted for age, markedly increased between 1990 and 2022, from 4.8% to 14.0% in men, and from 8.8% to 18.5% in women [1]. Obesity rates differ by race/ethnicity [1,2]. The proportion of adults with a body mass index (BMI) > 30 kg/m^2^ reaches 35% in the United States, and averages 15% in countries belonging to the Organization for Economic Co-operation and Development. In contrast, this proportion is relatively low in East Asian countries with an estimated 5% in Japan [2].

For healthcare providers, the increasing prevalence of obesity, particularly patients with extremely high body weight, means encountering situations where standard medical equipment is sometimes unavailable due to weight limitations [3]. Weight limitations of imaging equipment such as computed tomography (CT) scanners can lead to diagnostic delays, especially in emergency cases requiring immediate imaging evaluation [4]. This is compounded by the fact that healthcare staff is sometimes unaware of the precise weight and size specifications of the equipment available at the facility.

Herein, we present the case of a Caucasian male weighing 224.8 kg (BMI 73.4 kg/m^2^). The patient had type 2 diabetes and developed *Proteus mirabilis* bacteremia. A urinary tract infection (UTI) was diagnosed but CT imaging was initially not feasible due to equipment limitations related to the patient's excess body weight. Therefore, treatment had to proceed without excluding conditions requiring surgical intervention among UTIs. This case suggests the importance for healthcare providers of being familiar with the weight limits of medical equipment and preparing for cases in which patient weight exceeds them.

This case was previously presented as a meeting abstract in Japanese at the 61st Annual Meeting of the Japan Diabetes Society Kinki Regional Meeting on October 26, 2024.

## Case presentation

A 60-year-old Caucasian male patient presented to our outpatient clinic with a fever of 38.2 °C. Six days before the visit, he had developed a fever and sore throat. The patient had been diagnosed with pharyngitis and treated with acetaminophen and tranexamic acid; however, his fever persisted.

The patient had a height of 175 cm, weighed 224.8 kg, and his BMI was 73.4 kg/m^2^. He was conscious, with a blood pressure of 117/73 mmHg, a pulse rate of 117 beats/min, and blood oxygen saturation of 98% without ventilation. Auscultation of the lungs and heart revealed no abnormalities. The abdomen was soft and non-tender. There was no costovertebral angle tenderness or meningeal signs. The skin examination was notable only for mild bilateral lower extremity edema. Physical examination revealed no signs of endocrinological obesity, such as Cushing's syndrome or hypogonadism.

Regarding the course of his obesity, he had been born in the United States and developed obesity from a young age, weighing over 100 kg at the age of eight to nine. He came to Japan at the age of 20 as a foreign student of sumo wrestling, weighing 150 kg. He transitioned to coaching around the age of 26 and weighed 200 kg when he was around 30 years of age. At the age of 58, he reached a maximum weight of 260 kg and was also diagnosed with type 2 diabetes. He was treated with sitagliptin (50 mg/day), with a hemoglobin A_1C_ (HbA_1c_) level of 6-7%; however, his HbA_1c_ level had increased to 9-11% for six months before being admitted to our hospital.

Laboratory tests (Table 1) showed high levels of inflammatory response, with white blood cells at 23,900/μL and C-reactive protein (CRP) levels at 31.8 mg/dL. The patient presented with markedly elevated glycemic parameters, with HbA_1c_ and casual serum glucose levels being 11.8% and 631 mg/dL, respectively. Urinalysis revealed pyuria, with 10-19 leukocytes per high-power field. *P. mirabilis* was detected in both blood and urine cultures.

**Table 1:** Laboratory Findings Supporting the Diagnosis of Renal Infection. Abbreviations: WBC, White Blood Cell; Cr, Creatinine; eGFR, Estimated Glomerular Filtration Rate; CRP, C-Reactive Protein; HbA_1c_, Hemoglobin A_1c_; U-Ket, Urine Ketones; U-WBC, Urine White Blood Cells; HPF, High Power Field; U-TP, Urine Total Protein; FPG, Fasting Plasma Glucose; F-CPR, Fasting C-Peptide; GAD-Ab, Glutamic Acid Decarboxylase Antibodies; CysC, Cystatin C; eGFRcr, Estimated Glomerular Filtration Rate (creatinine-based); eGFRcys, Estimated Glomerular Filtration Rate (cystatin C-based).

Variable	Value	Reference range
Hematology		
WBC, /μL	23,900	3,300-8,600
Biochemistry		
Cr, mg/dL	1.94	0.65-1.07
eGFRcr, ml/min/1.73m^2^	29	>90
CRP, mg/dL	31.8	2.7-4.6
Casual Blood Glucose, mg/dL	631	Not fixed
HbA_1c_, %	11.8	4.9-6.0
Urinalysis		
U-Ket	(-)	(-)
U-WBC, /HPF	10-19	<5
U-TP, g/gCr	0.30	<0.15
Glucose-related tests (Day 4)		
FPG, mg/dL	217	73-109
F-CPR, ng/mL	4.07	1.1-3.3
GAD-Ab, U/mL	<5.0	<5.0
Biochemistry (Day 4)		
Cr, mg/dL	1.21	0.65-1.07
CysC, mg/L	1.58	0.61-1.00
eGFRcr, ml/min/1.73m^2^	48.6	>90
eGFRcys, ml/min/1.73m^2^	43.3	>90

Chest and abdominal radiographs revealed no abnormal lung shadows, and no gas or stones in the kidney area. Although other imaging studies were considered, his weight exceeded the maximum capacity of the CT scanners available at our facility (Brilliance iCT, 204 kg, Philips Healthcare, Amsterdam, Netherlands; Ingenuity, 204 kg, Philips Healthcare; and Aquilion Prime SP, 200 kg, Canon Inc., Tokyo, Japan). An ultrasound scan was not feasible due to the presence of thick subcutaneous fat.

The patient was diagnosed with a UTI, based on the detection of *P. mirabilis* from both blood and urine cultures and the fact that UTIs are its most common clinical manifestation [5]. Although a more detailed UTI diagnosis to determine the presence of abscesses, obstructive pyelonephritis, or urolithiasis should have been conducted [6], we were unable to do so because further imaging studies could not be carried out.

**Figure 1:**
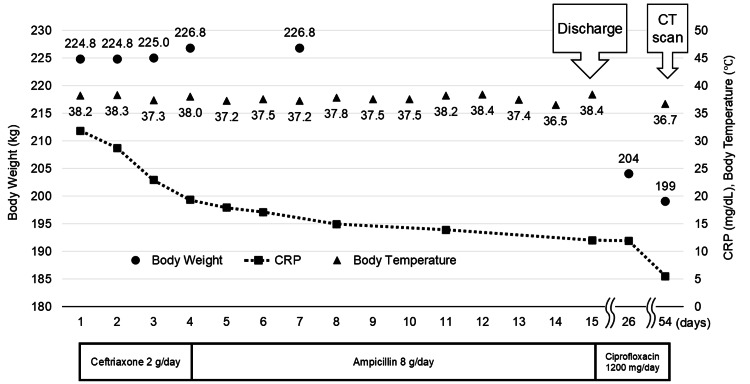
Clinical course. Body weight (circles), C-reactive protein (CRP; squares), and body temperature (triangles) during hospitalization and follow-up. Under antibiotics, the CRP levels showed slow but steady improvement, but the fever persisted. Computed tomography (CT) imaging could not be performed during hospitalization because the patient weight exceeded CT limits (204 kg). The need for combining individual beds caused discomfort in the patient and led to a request for early discharge. The patient was discharged on day 15. A CT scan was performed on day 54 when the patient weight (199 kg) had fallen below the maximum limit of the scanner. Weight was measured on the bed scale of the advanced care unit on days 1-4 and 7. After transfer to the general ward, weight measurements were impossible due to the maximum limit of the scale available at the ward (200 kg). Following discharge, the weight was recorded at outpatient visits on days 26 and 54.

Figure 1 shows the treatment course. Antibiotics treatment was started with ceftriaxone (2 g/day) and de-escalated to ampicillin (8 g/day). Under appropriate antibiotics for *P. mirabilis* infections, the C-reactive protein (CRP) levels showed slow but steady improvement, but the fever persisted.

To accommodate the patient, two beds were pushed together because the weight limit of the individual beds (138 kg) was exceeded. This caused sleep discomfort, and the patient requested early discharge. Despite the gradual clinical improvement, the patient was discharged on day 15 with oral antibiotics (ciprofloxacin 1200 mg/day).

Regarding glycemic management, the patient was fed a diet of 1,840 kcal per day. Continuous intravenous insulin was initially administered (rapid-acting insulin totaling up to 102 units per day), and as the symptoms of inflammation subsided, the patient was gradually switched to oral hypoglycemic agents. Semaglutide (3 mg/day) and metformin (500 mg/day) were administered, and the fasting levels of blood glucose were approximately 120 mg/dL.

**Figure 2:**
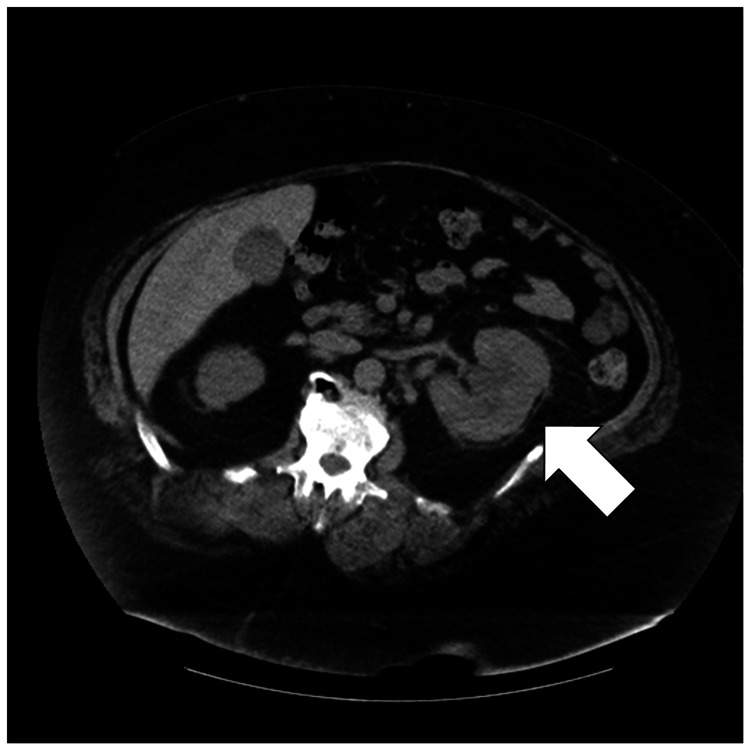
Post-discharge computed tomography (CT) imaging on day 54, performed after the patient weight had fallen below the weight limit of the CT equipment. Unenhanced CT showing a mildly enlarged left kidney and increased perirenal fatty tissue accumulation (arrow). The imaging findings were compatible with pyelonephritis, and given that the C-reactive protein levels had not normalized even after 54 days, progression to renal abscess was also considered probable. However, a definitive diagnosis could not be reached based on non-contrast CT imaging findings.

The patient lost weight after being discharged, and he was weighing 199 kg on day 54. Since this value was below the weight limit for the CT scan at our hospital, a scan was performed which revealed mild enlargement of the left kidney along with increased perirenal fatty tissue accumulation (Figure 2). The imaging findings were compatible with pyelonephritis, and since the CRP levels had not normalized even after 54 days, progression to renal abscess was also considered probable; however, the definitive diagnosis could not be established by non-contrast CT imaging. Nevertheless, considering the gradual improvement in CRP levels and the absence of fever, the diagnosis of a healed renal infection was confirmed. The clinical course of the patient was successful, and antibiotics were discontinued at that time. Six months after discharge, the patient was in good condition and weighed approximately 190 kg.

## Discussion

We report a case of a patient treated for a UTI with bacteremia whose extremely high body weight initially prevented an imaging-based diagnosis. A post-discharge CT confirmed the diagnosis of a renal infection. The clinical course provides insights into the management of patients with obesity, and specifically of the importance of being aware of the exact weight limits of hospital instruments and the location of nearby high-capacity CT scanners.

The first lesson is that it is important to be aware of the weight and size limits of the medical instruments available at a particular hospital. Older CT systems have a weight limit of 205 kg, while the more recent ones can accommodate patients weighing up to 308 kg [4]. Exceeding these limits can lead to failure in the table movement mechanism of the scanner [4].

We initially believed that MRI equipment had stricter weight limitations compared to CT. Indeed, typical MRI scanners have a lower weight limit compared to typical CT scanners (158 kg vs. 204 kg) [7,8]. Therefore, we assumed that MRI would be impossible to use in this particular case, since the patient weight (224.8 kg) exceeded the maximum weight limit of our CT scanner (204 kg). However, investigation after discharge revealed that the MRI scanner available at our facility had a weight capacity of 250 kg. This case highlights the need for updated knowledge of the evolving medical equipment specifications to ensure optimal diagnosis. Nevertheless, the condition of the patient showed slow but steady improvement following antibiotic treatment, suggesting a positive response and decreasing the urgency for imaging data. Even if MRI information had been possible to obtain, it would not have changed the clinical course, since the patient recovered without surgical intervention. In addition, MRI might still not have been possible due to the bore diameter of the scanner (70 cm), which may not have been suitable.

Second, healthcare staff should be aware of the locations of nearby high-capacity CT scanners to optimize the management strategy adopted for patients with extremely high body weight [4]. For example, some CT scanners (CT 7500, Philips Healthcare, Amsterdam, Netherlands, and Aquilion ONE, Canon Inc., Tokyo, Japan) equipped with optional high-capacity beds can be used to perform scans on patients weighing up to 307 kg and 300 kg, respectively. However, we did not have at the time information about nearby high-capacity CT scanners. In agreement with our experience, a report from England indicates that 76% of medical facilities are unaware of where to transfer patients exceeding the maximum weight or size capacity of their CT scanners [4].

The inability to perform timely CT imaging impacted the diagnosis and treatment processes. We could not rule out conditions requiring surgical intervention among UTI, such as urolithiasis, during the treatment course. Consequently, we were forced to treat the renal infection without imaging, which fortunately resulted in clinical resolution.

In general, for emergency conditions such as stroke or aortic dissection, timely imaging is crucial [4]. We have performed a systematic search in the PubMed database comprising the past 10 years (January 2014 - November 2024), and found 10 reported cases with accessible abstracts and/or full texts (including ours) where imaging tests could not be performed due to the weight or size limits of the imaging equipment [3,9-16]. These reports originated from various countries: the United States (four cases) [10,11,13,15], France (three cases) [12,14,16], Japan (two cases, including ours) [3], and Canada (one case) [9]. In nine out of 10 cases, the initial diagnosis of the patient was critical (three cases of pulmonary embolism [11,12,16], two of sepsis [3,14], one of stroke [9], one of aortic dissection [15], one of bacteremia (our case), and one of common bile duct obstruction [13]). While publication bias cannot be ruled out, these cases suggest that timely imaging is often needed in emergencies involving severe obesity.

Utilizing existing medical resources offers an effective approach to improve healthcare delivery systems for extremely high-weight patients. In East Asian regions, particularly Japan, where patients over 200 kg are uncommon, upgrading individual facilities is cost-prohibitive. A policy of identifying and publicizing facilities with specialized equipment - including high-capacity beds, operating tables, and imaging systems - could provide a practical solution and reduce delays in inter-hospital communication, which can be life-threatening for patients [4]. Medical staff also should be prepared for the additional challenges involved in patient transport within and between facilities, such as ensuring that appropriate passageways are available [7]. Therefore, a protocol is needed to avoid delays in accessing imaging equipment for patients with extremely high body weight.

## Conclusions

We report a case of *P. mirabilis* bacteremia and renal infection in a patient with severe obesity and type 2 diabetes. We treated the patient in the absence of CT imaging data, and a post-discharge CT scan performed when his weight fell below the 204 kg limit of the CT scanner showed a mildly enlarged left kidney and increased perirenal fatty tissue accumulation compatible with a healed pyelonephritis or renal abscess. With the increasing prevalence of obesity worldwide and the growing cross-border movement of people, it is likely that patients with extremely high body weight requiring medical imaging will become more common. Weight restrictions on medical equipment can delay diagnoses, especially in emergencies such as those involving stroke or aortic dissection, and this is compounded by the limited awareness of equipment specifications among healthcare providers.

This case demonstrates several lessons for clinical practice. First, the maximum body weight limit of the available imaging equipment should be known in advance. MRI may be an alternative for patients who exceed the weight limits of the CT equipment in some facilities. Additionally, nearby medical facilities with high-capacity imaging equipment where patients with these characteristics could be transferred should be properly identified.
